# The Efficacy and Safety of Infliximab in Refractory Noninfectious Uveitis: A Meta-Analysis of Observational Studies

**DOI:** 10.3389/fphar.2021.620340

**Published:** 2021-09-16

**Authors:** Anji Xiong, Deng Liu, Huini Chen, Guancui Yang, Chen Xiong, Yu Shuai, Linqian He, Zepeng Guo, Liangwen Zhang, Yuan Yang, Beibei Cui, Shiquan Shuai

**Affiliations:** ^1^ Department of Rheumatology and Immunology, Nanchong Central Hospital, The Second Clinical Medical College, North Sichuan Medical College, Nanchong, China; ^2^ Inflammation and Immunology Key Laboratory of Nanchong, Nanchong, China; ^3^ Department of Rheumatology and Immunology, The First Affiliated Hospital of Chengdu Medical College, Chengdu, China; ^4^ Department of Rheumatology and Immunology, West China Hospital, Sichuan University, Chengdu, China

**Keywords:** infliximab, noninfectious uveitis, anti-TNF-α, uveitis treatment, refractory

## Abstract

**Background:** Although infliximab has been recommended for the second-line treatment of seronegative spondyloarthropathy- or juvenile idiopathic arthritis-related uveitis, the issue of its systemic efficacy and safety in a broader diversity of refractory noninfectious uveitis is debatable. To assess the short-term and relatively long-term efficacy of infliximab in refractory noninfectious uveitis, we performed a systematic review and meta-analysis of observational studies.

**Methods:** PubMed, Cochrane Library, EMBASE, and Wanfang Med Online were systematically searched from January 2005 to March 2020. Two investigators independently assessed eligibility. Data were independently collected by two investigators. The pooled proportions were estimated with patients for intraocular inflammation control and improvement of visual acuity. Pooled proportions with 95% credible intervals were computed. Study homogeneity was investigated using *I* ^2^ statistics to quantify the percentage of variation across studies. To pool the results, the Mantel–Haenszel fixed-effects or random-effects models were used.

**Results:** Of 2316 studies identified, 16 unique studies with 509 unique participants were included in the meta-analysis. The pooled proportions of intraocular inflammation control reached 92% (95% CI: 87%–98%; *I* ^2^: 1%; p=0.42) and 95% (95% CI: 93%–97%; *I* ^2^: 0%; p=0.91) in groups of ≤6- and ≥12-month follow-up durations. During the relatively long follow-up period, the pooled proportions of maintaining visual acuity stable or increasing at least one line reached 99% (95% CI: 96%–100%; *I* ^2^: 0%; p=0.54) in the involved eyes. The corticosteroid-sparing effect of infliximab was also well demonstrated, with the proportion of corticosteroid-sparing success reaching 85.5% (112/131). Besides, about serious adverse events, 2.6% (13/500) of patients experienced hypersensitivity reactions, 2.4% (12/500) of patients experienced serious infections, 1.8% (9/500) of patients experienced autoimmune diseases, and 0.6% (3/500) of patients experienced neoplasia.

**Conclusions:** This meta-analysis provided evidence that infliximab might be a promising choice in controlling inflammatory activity, gaining visual acuity, and sparing corticosteroid use with relatively few side effects when applied in treating refractory noninfectious uveitis.

**Systematic Review Registration**: [website], identifier [registration number]

## Introduction

Noninfectious uveitis is a group of various etiologies-related sight-threatening inflammatory diseases which affects the iris, ciliary body, vitreous, retina, and choroid(Nussenblatt, 1990; Duica et al., 2018; Krishna et al., 2017; Trivedi and Katelaris, 2019). It has been estimated as the third leading cause of blindness in the world (Lee, 2015; de Parisot et al., 2017), responsible for 5–10% of visual impairment globally (Miserocchi et al., 2013; Tsirouki et al., 2018). Conversely, up to 35% of patients with uveitis suffer from significant visual loss to legal blindness (Munoz-Fernandez et al., 2006; de Smet et al., 2011). To limit potentially sight-threatening complications, good control of the inflammation in the acute phase is necessary (Toguri et al., 2018; Wildner and Diedrichs-Mohring, 2019). Currently, corticosteroids have been the mainstay of therapy for noninfectious ocular inflammatory disease (LeHoang, 2012; Rossi et al., 2019; Ormaechea et al., 2019; Jabs et al., 2000). However, in cases of refractory uveitis, corticosteroids therapy still has certain limitations (Riancho-Zarrabeitia et al., 2015; Duica, 2018; Koronis et al., 2019). Notably, corticosteroids alone are not sufficient for the treatment of many cases of chronic uveitis and do not prevent further relapses (Kruh and Foster., 2012; Jabs, 2018). Additionally, long-term administration of corticosteroid may result in unacceptable side effects including Cushingoid changes, iatrogenic diabetes, osteoporosis, cataract formation, increased intraocular pressure (IOP), and hormone-related glaucoma (Levy-Clarke et al., 2014; Rice et al., 2017; Oray et al., 2016). To prevent irreversible structural damage and blindness, other forms of immunosuppressive therapy are warranted (Tomkins-Netzer et al., 2012; Pasadhika and Rosenbaum, 2014). Infliximab (IFX) is a monoclonal chimeric IgG1 antibody designed to intercept and neutralize tumor necrosis factor-alpha, a key inflammatory cytokine (Lichtenstein et al., 2015; Radner and Aletaha, 2015; Horiuchi et al., 2010). Recently, emerging evidences have shown that IFX is moderately or highly efficacious in suppressing uveitis, allowing a significant reduction in the mean corticosteroid dose (Petiti Martin et al., 2012). But, until now, IFX has been recommended as the second-line immunomodulatory agent in treating seronegative spondyloarthropathy- or juvenile idiopathic arthritis-related uveitis (Levy-Clarke et al, 2014; Angeles-Han et al., 2019; Hatemi et al., 2019). In fact, besides them, many other diseases also can be the etiologies of noninfectious uveitis (Rosenbaum and Dick, 2018), such as birdshot retinochoroidopathy, sarcoidosis, Vogt–Koyanagi–Harada syndrome, Behcet’s disease, juvenile-onset rheumatological disease, rheumatoid arthritis, relapsing polychondritis, Crohn’s disease, psoriasis, and mucous membrane pemphigoid. Besides, there is a kind of noninfectious uveitis with no obvious underlying etiology, that is, idiopathic uveitis. Therefore, the efficacy of IFX in a broader diversity of refractory noninfectious uveitis also deserves our attention.

At present, IFX has already been recommended for the second-line treatment of seronegative spondyloarthropathy- or juvenile idiopathic arthritis-related uveitis (Levy-Clarke, 2014; Angeles-Han et al, 2019; Hatemi et al, 2019). Nevertheless, the clinical evidence or expert recommendation for IFX in a broader diversity of refractory noninfectious uveitis such as birdshot retinochoroidopathy, sarcoidosis- or rheumatoid arthritis-related uveitis, and idiopathic uveitis is still lacking.

We herein performed a meta-analysis and review aimed at systematically synthesizing the previous clinical evidence and evaluating the efficacy and safety of IFX in the treatment of a broader diversity of refractory noninfectious uveitis.

## Methods

### Eligibility Criteria

The following criteria were used in the selection of studies for review: 1) noninfectious autoimmune uveitis-related studies (separate studies of seronegative spondyloarthropathy- or juvenile idiopathic arthritis-related uveitis were excluded to avoid potential bias); 2) the uveitis inflammatory activity grading defined based on the Standardization of Uveitis Nomenclature (SUN) working-group criteria (Jabs et al., 2005); 3) patients with refractory uveitis which was considered persistently active for at least 3 months despite previous systemic steroids and/or immunosuppressive treatment; 4) the follow-up duration was at least 3 months; and 5) studies with at least five patients to avoid a positive report bias.

### Outcome Measures

1) Control of intraocular inflammation was defined by the anterior chamber cells and/or vitreous haze decreasing by two levels or to grade 0.5 + or 0, according to the SUN criteria and National Eye Institute system criteria adopted by SUN in at least one eye;

2) controlled visual acuity (VA), according to the SUN criteria (Jabs et al, 2005), was defined as a doubling of the visual angle (converted into a logMAR format) in the involved eye from the baseline (corresponding to an increase of three lines on a decimal scale with a logarithmic chart). In addition, we also collected information of VA maintaining stable or improving at least one line;

3) corticosteroid-sparing success was defined as an inactive inflammation after tapering corticosteroid (topical, periocular, oral, or intravenous) to 10 mg daily or less;

4) treatment failure was defined as the SUN-cell-activity score (anterior chamber cell/vitreous haze grade) had worsened (a two-grade increase) or had not decreased to ≤0.5 +; and

5) safety was mainly assessed by the occurrence of serious adverse events (SAEs), which included autoimmune diseases, neoplasia, hypersensitivity reactions, and serious infections. SAEs were specified as IFX treatment interruption due to unacceptable side effects. In addition, we also analyzed the incidence of common minor adverse events (MAEs).

### Sources and Search Methods

The literature search for review was conducted using the electronic databases of published studies (i.e., PubMed, Cochrane Library, EMBASE, and Wanfang Med Online) from January 2005 to March 2020, with language restriction of only articles in English. Search algorithms included the following MESH terms: (“Infliximab” or “Tumor Necrosis Factor-alpha” or “Tumor Necrosis Factor alpha” or “Cachectin” or “Cachectin-Tumor Necrosis Factor” or “Cachectin Tumor Necrosis Factor” or “Tumor Necrosis Factor Ligand Superfamily Member 2” or “Tumor Necrosis Factor” or “TNF Superfamily, Member 2” or “TNFalpha” or “TNF-alpha”) and (“Uveitis” or “Uveitides”). No restrictions were made on uveitis such as using the words “chronic,” “anterior,” “posterior,” “infectious,” “noninfectious,” and “autoimmune” to expand the number of hits in the literature to be screened. Uncontrolled case series, nonrandomized, retrospective clinical studies, and prospective open-label trials were included to provide evidences related to the effectiveness of IFX in uveitis ultimately, as there are no randomized controlled trials on the treatment of noninfectious uveitis with IFX up to now.

### Study Selection and Data Collection

**FIGURE 1:**
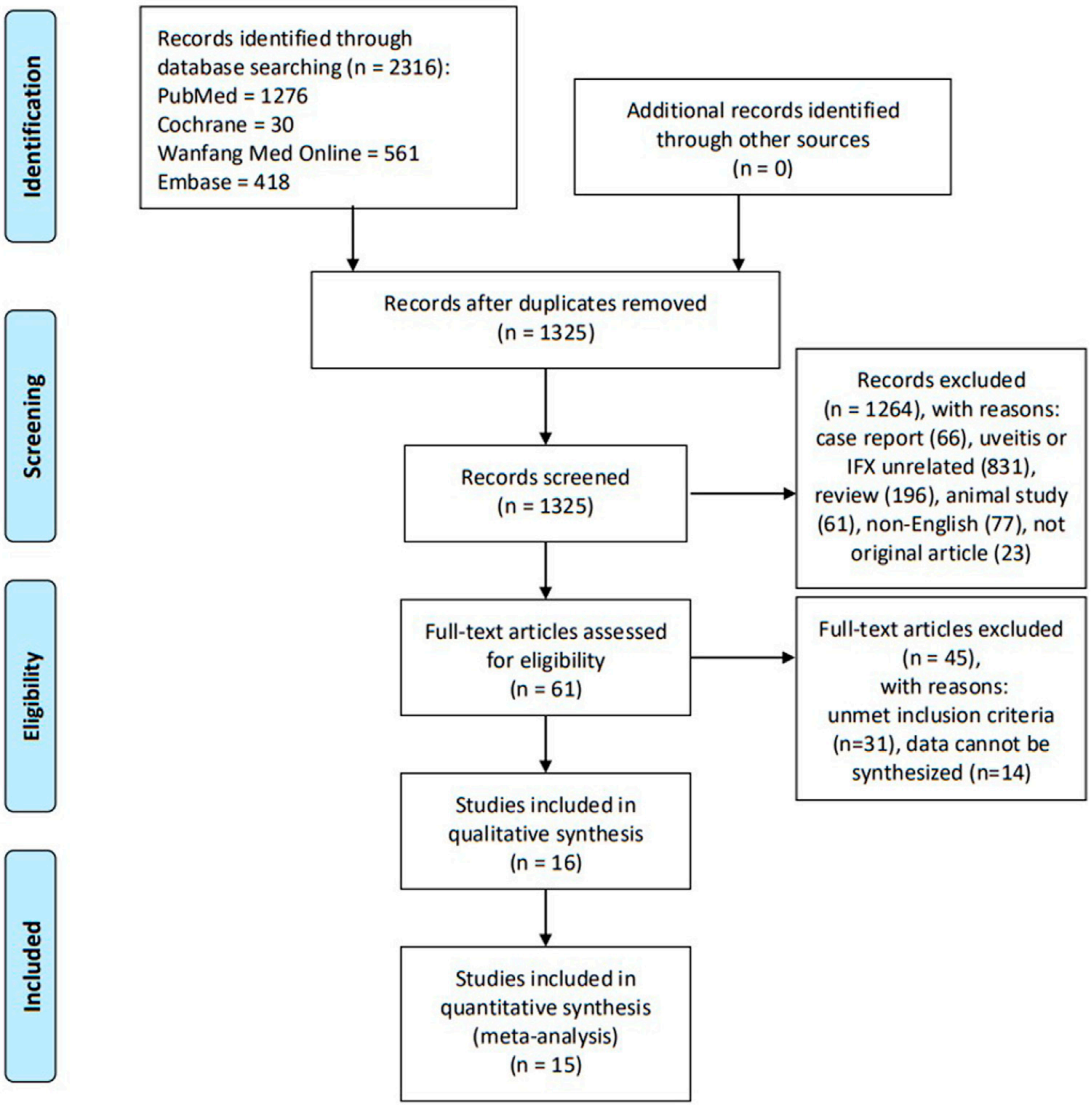
Flowchart demonstrating the process of study selection.

Three reviewers (Deng Liu, Anji Xiong, and Huini Chen) independently screened the titles and abstracts of the searched studies and determined their relevance to this meta-analysis. Full-text articles were retrieved and assessed for eligibility. Evaluations of methodological quality and risk of bias were undertaken. Any disagreements were resolved through discussion until a consensus was reached. Key information gathered from the selected articles was listed in a standard form containing relevant details: study design type, number of patients, age and gender statistics, follow-up duration, and definitions of outcomes and results. If the same registered trial appeared on sequential or multiple publications, the data from the most recent or comprehensive publication were included. A flow diagram (Figure 1) was used to illustrate the details of the selection process including reasons for the exclusion of articles.

### Quality Assessment

The methodological quality of the studies included was assessed using an 11-item checklist which was recommended by Agency for Healthcare Research and Quality (AHRQ). An item would be scored “0” if it was answered “NO” or “UNCLEAR”; if it was answered “YES,” then the item scored “1.” Article quality was assessed as follows: low quality = 0–3, moderate quality = 4–7, and high quality = 8–11. The risk of bias was assessed independently using the AHRQ risk-of-bias tool. Each of the eleven items was classified as having low, medium, or high risk of bias. Low quality is considered as high risk; moderate quality is considered as medium risk; and high quality is considered as low risk.

### Data Synthesis and Analysis

Description of outcomes was graded dichotomously as “yes” or “no” responses. Studies reported their outcomes by diverse analytical methods, either based on time points (e.g., 6, or 12 months) or based on the median follow-up durations, with different analytical methods. For the analysis of the pooled proportion of controlled intraocular inflammatory activity, extracted data were categorized into two groups of “6 months or less” and “12 months or more” and this grouping helped explain the short-term and relatively long-term efficacy of IFX on uveitis, respectively. The strength of evidence of nonrandomized clinical trials was rated using a scale from the Oxford Centre for Evidence-Based Medicine. All assessments were independently done by three investigators (Deng Liu, Anji Xiong, and Huini Chen).

This meta-analysis was conducted in concordance with the MOOSE guidelines for systematic reviews and meta-analyses (Stroup et al., 2000). The pooled proportions and 95% CIs were realized by the “Metaprop” program package in R 3.6.3. Study homogeneity was investigated using *I* ^2^ statistics to quantify the percentage of variation across studies. A random-effects model (DerSimonian–Laird method) was employed when the *I* ^2^ ＞50% and *p* ＜ 0.1; otherwise, a fixed-effects model (Mantel–Haenszel method) was used. Subgroup analysis was conducted if obvious heterogeneity existed. The difference in groups of pooled proportions was statistically significant when *p* ＜ 0.05. A meta-analysis of clinical trials was done with a similar statistical procedure, if possible; otherwise, a systematic review was conducted. Potential publication bias was assessed by Egger’s test and presented in funnel plots. When a few studies are included in the analysis, the power of the tests is too low; therefore, publication bias was only examined if more than 10 study comparisons were included in the analysis.

## Results

### Selection of Studies

A systematic search of multiple electronic databases yielded a total of 2,316 possible relevant articles; of these, 2,255 were excluded after scanning the titles and abstracts. After full-text scrutiny of the remaining 61 articles, 31 articles were excluded due to unmet inclusion criteria. Thereafter, 14 potentially eligible articles were removed because their data type could not be synthesized together. Finally, 16 studies were retained for the meta-analysis. A flow diagram (Figure 1) was used to illustrate the details of the selection process including reasons for the exclusion of articles.

### Characteristics of Included Studies

The characteristics of the selected studies are summarized in Supplementary Table S1; (Sharma et al., 2007; Sobrin et al., 2007; Simonini et al., 2008; Tugal-Tutkun et al., 2008; Giardina et al., 2011; Simonini et al., 2011; Martel et al., 2012; Pichaporn et al., 2013; Kruh et al., 2014; Lian et al., 2015; Vallet et al., 2015; Vallet et al., 2016; Mercier et al., 2018; Noy et al., 2019; Sharma et al., 2019; Yalçindag and Köse, 2020). All of these studies were observational, nonrandomized case series and had a median or mean follow-up duration of more than 6 months. In all studies, 5–10 mg/kg of infliximab was infused at weeks 0, 2, and 6 and then every 4–8 weeks. The infusion frequency and dose were depending on the indication for therapy and disease activity. One study had 3a evidence strength, three studies had 2b evidence strength, and the other twelve studies had four evidence strength according to the Oxford Centre for Evidence-Based Medicine (March 2009).

### Risk-of-Bias Assessment

**FIGURE 2:**
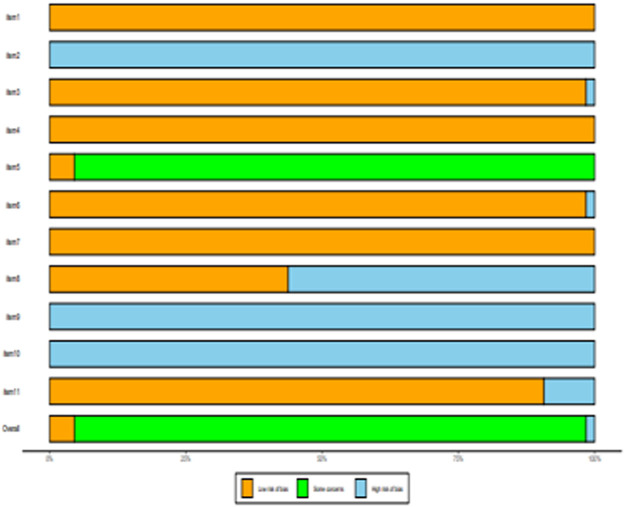
Risk-of-bias summary of included studies using the AHRQ risk-of-bias tool.

All of the selected articles were assessed for methodological quality. The results of the quality assessment are shown in Supplementary Table S2. One study was of high quality and fourteen studies were of moderate quality. There was one article with low quality rating. The risk for bias for the included studies is presented in Figure 2.

### Control of Intraocular Inflammation

**FIGURE 3:**
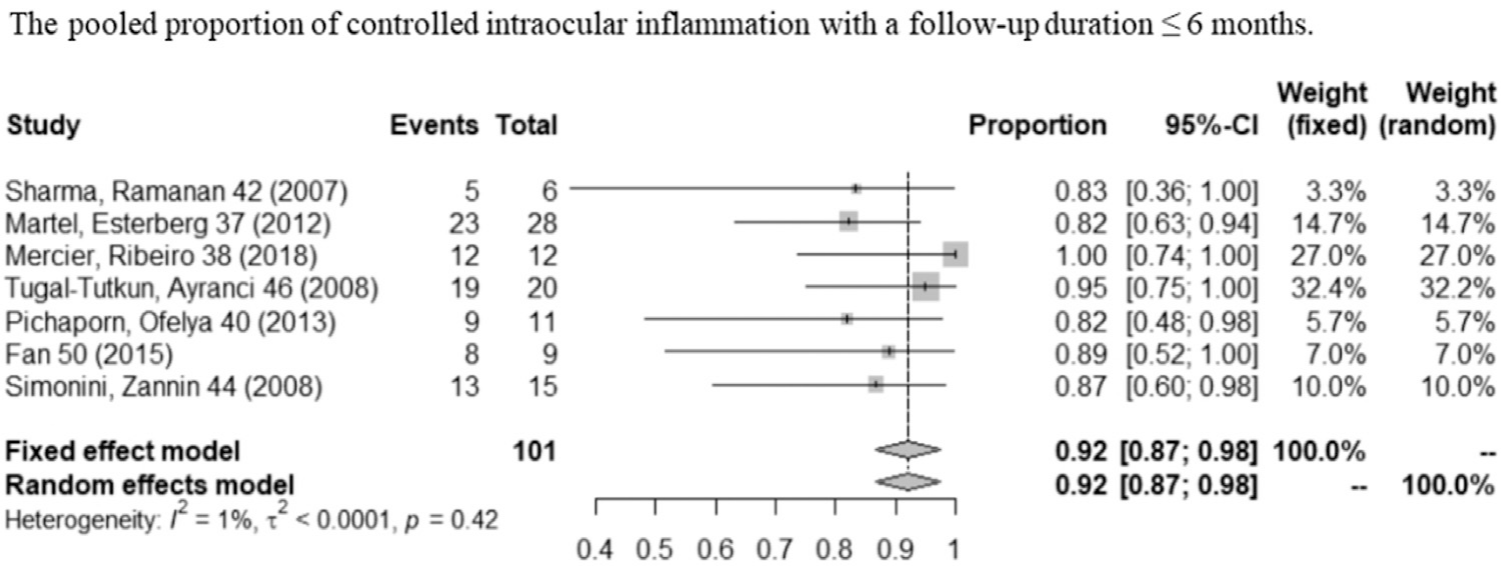
Pooled proportion of controlled intraocular inflammation with a follow-up duration of ≤6 months.

**FIGURE 4:**
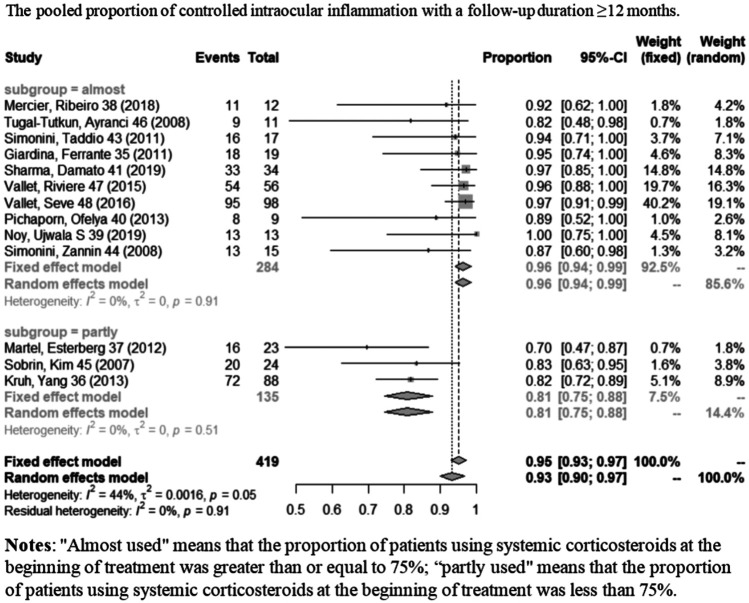
Pooled proportion of controlled intraocular inflammation with a follow-up duration of ≥12 months.

A total of 16 studies showed controlled intraocular inflammation matching our criteria, of which two (Sharma et al, 2007; Lian et al, 2015) only contained information of follow-up time ≤6 months; nine (Sobrin et al, 2007; Giardina et al, 2011; Simonini et al, 2011; Kruh et al, 2014; Vallet et al, 2015; Vallet et al, 2016; Noy et al, 2019; Sharma et al, 2019; Yalçindag and Köse, 2020) studies only contained information of follow-up time ≥12 months; and five (Simonini et al, 2008; Tugal-Tutkun et al, 2008; Martel et al, 2012; Pichaporn et al, 2013; Mercier et al, 2018) contained both. Figure 3 shows the pooled proportion results of controlled intraocular inflammation in a meta-analysis. When the follow-up duration was ≤6 months, the pooled controlled intraocular inflammation proportion was 92% (95% CI: 87%–98%; *p* = 0.42), with no statistically significant difference compared to a follow-up duration of ≥12 months (95%, 95% CI: 93%–97%; *p* = 0.91). As shown in Figure 4, with obvious heterogeneity in the combination of proportions with follow-up ≥ 12 months (*I* ^2^ = 44%, *p* < 0.01), a subgroup analysis was conducted by dividing studies into “almost used” (≥75% of the patients used systemic CS) and “partly used” (＜75% of the patients used systemic CS) according to systemic corticosteroid usage during IFX therapy. When systemic CS was used as “almost used,” the pooled proportion of intraocular inflammation control reached the highest value (96%, 95% CI: 94%–99%; *p* = 0.91). Heterogeneity was well resolved after subgroup analysis.

### Visual Acuity

**FIGURE 5:**
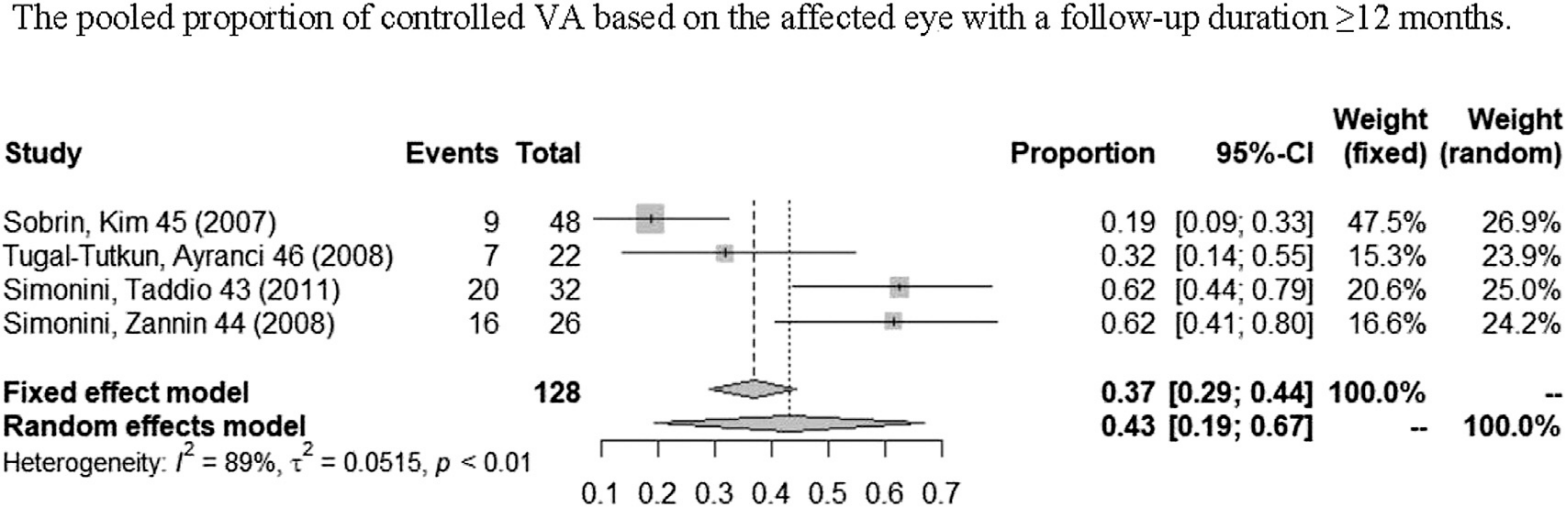
Pooled proportion of controlled VA based on the affected eye with a follow-up duration of ≥12 months.

**FIGURE 6:**
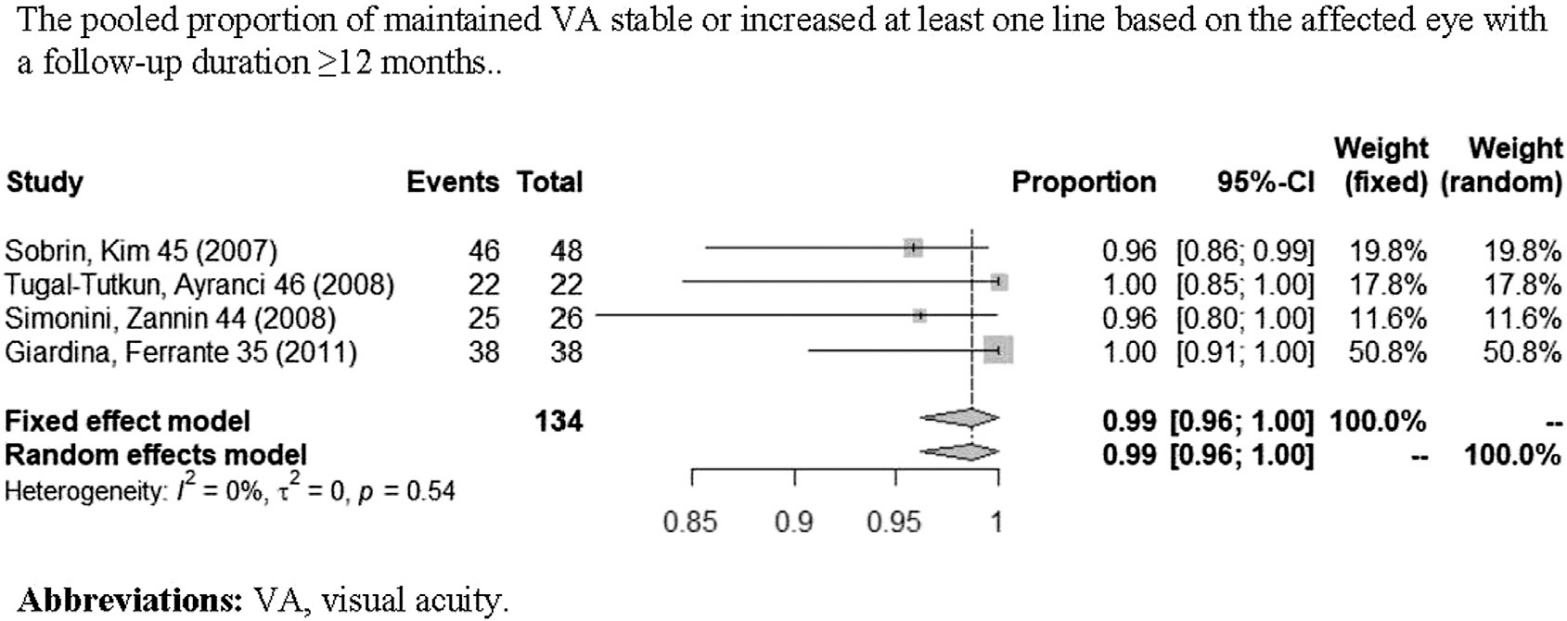
Pooled proportion of maintained VA stable or increased at least one line based on the affected eye with a follow-up duration of ≥12 months.

A total of nine studies (Sharma et al, 2007; Sobrin et al, 2007; Simonini et al, 2008; Giardina et al, 2011; Simonini et al, 2011; Vallet et al, 2016; Noy et al, 2019; Sharma et al, 2019; Yalçindag and Köse, 2020) reported VA outcomes. One study (Pichaporn et al, 2013) only contained information of visual acuity change based on per affected eye, two studies (Giardina et al, 2011; Lian et al, 2015) only contained information of visual acuity change based on per patient, and five studies (Sharma et al, 2007; Sobrin et al, 2007; Simonini et al, 2008; Tugal-Tutkun et al, 2008; Simonini et al, 2011) contained both. Figure 5 shows the pooled proportion results of visual acuity improvement with a long-term follow-up (≥12 months) in a meta-analysis. As shown in Figures 5, the pooled proportion of controlled VA reached 43% (95% CI: 19%–67%; *p* < 0.01) in the involved eyes (Sobrin et al, 2007; Simonini et al, 2008; Tugal-Tutkun et al, 2008; Simonini et al, 2011), and the pooled proportion of VA remaining stable or improving at least one line reached 99% (95% CI: 96%–100%; *p* = 0.54) in the involved eyes Figure 6 (Sobrin et al, 2007; Simonini et al, 2008; Tugal-Tutkun et al, 2008; Giardina et al, 2011). In addition, in one study (Yalçindag and Köse, 2020), a total of 20 patients reported a significant improvement of BCVA (best-corrected visual acuity) after 1 year of IFX treatment when compared with pretreatment.

### Corticosteroid-Sparing

Information on corticosteroid-sparing was extractable in eight of the sixteen studies. 82.9% (68/82) of patients completely discontinued corticosteroids. Additionally, 85.5% (112/131) of patients achieved corticosteroid-sparing success. Details are recorded in Supplementary Table S1.

### Safety

Fifteen studies (Sharma et al, 2007; Sobrin et al, 2007; Simonini et al, 2008; Tugal-Tutkun et al, 2008; Giardina et al, 2011; Simonini et al, 2011; Martel et al, 2012; Pichaporn et al, 2013; Kruh et al, 2014; Vallet et al, 2015; Vallet et al, 2016; Mercier et al, 2018; Noy et al, 2019; Sharma et al, 2019; Yalçindag and Köse, 2020) provided information on serious adverse events (SAEs). 2.6% (13/500) of patients experienced hypersensitivity reactions, 2.4% (12/500) of patients experienced serious infections, 1.8% (9/500) of patients experienced autoimmune diseases, and 0.6% (3/500) of patients experienced neoplasia. In addition, 11.2% (54/481) of patients reported minor adverse events (MAEs) in fourteen studies (Sharma et al, 2007; Sobrin et al, 2007; Simonini et al, 2008; Tugal-Tutkun et al, 2008; Simonini et al, 2011; Martel et al, 2012; Pichaporn et al, 2013; Kruh et al, 2014; Vallet et al, 2015; Vallet et al, 2016; Mercier et al, 2018; Noy et al, 2019; Sharma et al, 2019; Yalçindag and Köse, 2020). The most frequently reported MAEs were minor infections (2.9%, 14/481), skin rash (1.9%, 9/481), fatigue (1.5%, 7/481), and injection-site reactions (1.0%, 5/481). Details are recorded in Supplementary Table S1.

### Treatment Interruption

A total of 14 studies (Sharma et al, 2007; Sobrin et al, 2007; Simonini et al, 2008; Tugal-Tutkun et al, 2008; Simonini et al, 2011; Martel et al, 2012; Pichaporn et al, 2013; Kruh et al, 2014; Vallet et al, 2015; Vallet et al, 2016; Mercier et al, 2018; Noy et al, 2019; Sharma et al, 2019; Yalçindag and Köse, 2020) had the information of IFX discontinuation. Details are also recorded in Supplementary Table S1. 13.7% (55/402) of patients discontinued IFX treatment, of which 8.2% (33/402) of patients discontinued IFX for SAEs, and 5.5% (22/402) of patients discontinued IFX for other reasons.

### Publication Bias

Funnel plot results for potential publication bias are shown in Supplementary Figure S1. From visual inspection of the funnel plot, there was pronounced asymmetry denoting publication bias. The Egger test for funnel plot asymmetry was significant in the proportion of intraocular inflammation control in long term (≥12 months) (*p* = 0.009). Other outcome indicators were observed for less than ten studies, so publication bias was not examined.

## Discussion

We systematically reviewed many aspects of the efficacy of IFX in refractory noninfectious uveitis including control of intraocular inflammation, improvement of visual acuity, and corticosteroid-sparing. In addition, we assessed safety by the occurrence of serious adverse events (SAEs) which included autoimmune diseases, neoplasia, hypersensitivity reactions, and serious infections. We defined the controlled intraocular inflammation by the anterior chamber cells and/or vitreous haze decreasing by two levels or to grade 0.5 + or 0, according to the SUN criteria and National Eye Institute system criteria adopted by SUN in at least one eye. Perhaps the pooled proportion of controlled intraocular inflammation based on each eye can better explain the curative effect of IFX on affected eyes, but in fact, detailed information about inflammation control of affected eyes is lacking and unavailable in most studies. Considering the diversity of etiologies of uveitis included, the etiologies of patients and the corresponding number were documented in detail in Supplementary Table S1. We calculated the percentage of patients with seronegative spondyloarthropathy- or juvenile idiopathic arthritis. According to rough calculation, the patients with seronegative spondyloarthropathy- or juvenile idiopathic arthritis-related uveitis accounted for about 1/5. Therefore, uveitis related to these etiologies has a limited impact on statistical results. The efficacy of IFX in the treatment of a broader diversity of refractory noninfectious uveitis is still well proved.

First of all, we divided the endpoint into two subgroups to examine the short-term (≤6 months) and relatively long-term (≥12 months) effects on inflammation control. In our combined analysis of short-term and relatively long-term follow-up periods, we found the pooled proportions of intraocular inflammation control reached 92% (95% CI: 87%–98%) and 95% (95% CI: 93%–97%), respectively, indicating that stable therapeutic effect can be obtained. We found obvious heterogeneity in the combination of proportions with follow-up ≥ 12 months (*I* ^2^ = 44%, *p* < 0.01), and thus, a subgroup analysis was conducted according to the patient proportion of using systemic corticosteroids during IFX treatment. After the subgroup analysis, heterogeneity was well resolved. Therefore, the subgroup analysis indicated the “almost used” of CS contributed to such heterogeneity exited. When the subgroup was “almost used,” the pooled proportion of activity control reached 96% (95% CI: 94%–99%; *p* = 0.91) which was better than the “partly used” (81%, 95% CI: 75%–88%; *p* = 0.51). Comparing the results of these two subgroups can help us to judge that IFX combined with CS may have a better therapeutic effect and can control inflammation more quickly. Therefore, in order to gain a better long-term efficacy (≥12 months) on inflammation control, early combined application of IFX and CS may be worth recommending.

In the short-term follow-up group (≤6 months), we did not conduct a subgroup analysis according to the patient proportion of using systemic corticosteroids because almost no heterogeneity was observed, and more importantly, the information on CS use ratio in short-term follow-up was also lacking and unextractable. Thus, we cannot explain whether it will be a better short-term efficacy in intraocular inflammation control when choosing an early combination therapy of IFX and CS in noninfectious uveitis. However, we still strongly recommend the early combination of IFX and CS in uveitis in order to obtain a better long-term curative effect and prevent progressive irreversible damage to the eyes.

We analyzed the results of visual acuity change based on the affected eye. According to the meta-analyses, respectively, the pooled proportion of controlled VA based on the affected eye is 43% (95% CI: 19%–67%). But this result existed obvious heterogeneity. The main reason may be that in some involved eyes, the structural damage was already present before IFX therapy, which caused VA that can only be maintained in a stable state instead of increasing further. Therefore, we analyzed the pooled proportion of maintaining VA stable or increasing at least one line. According to the outcomes of meta-analysis, the pooled proportion of maintaining stable VA or increasing at least one line based on the affected eye was 99% (95% CI: 96%–100%; *p* = 0.54) with no heterogeneity observed (*I* ^2^ = 0%), which indicates a significant effect of IFX in maintaining VA stability and even improving. Although there is a limited reference value for the pooled proportion of controlled VA due to its significant heterogeneity, the good efficacy of IFX in maintaining VA stability and even improving is still well proved in this meta-analysis.

We briefly analyzed the corticosteroid-sparing effect. According to the extractable information, in eight studies, 82.9% (68/82) of patients completely discontinued corticosteroid and 85.5% (112/131) of patients achieved corticosteroid-sparing success. We did not conduct meta-analysis for the corticosteroid-sparing effect because the number of studies which can extract corticosteroid-sparing information is too few. Besides, some studies which take corticosteroid discontinuation as the success criterion of corticosteroid-sparing have not included patients who still maintain low-dose corticosteroid (≤10 mg/ day). Although the corticosteroid-sparing effect was simply analyzed by averages without considering the weight of each study, the outcomes also indicated that IFX may have a good corticosteroid-sparing effect. In addition, such a significant corticosteroid-sparing effect also avoided adverse events induced by corticosteroids during uveitis activity control. According to the simple analysis of the incidence of side effects, some serious adverse events such as autoimmune diseases, neoplasia, hypersensitivity reactions, and serious infections also should be noted when using IFX to treat noninfectious uveitis, although they have only a low incidence.

Above all, several promising results have contributed to the reasonable use of IFX in the clinical treatment of refractory uveitis. 1) IFX may have a stable inflammation control effect in the short-term and relatively long-term treatment of uveitis. 2) An early combination of IFX and systemic CS can obtain better long-term efficacy in inflammation control of uveitis. 3) IFX can effectively control the worsening of VA. 4) IFX has a good corticosteroid-sparing effect. 5) IFX was generally well tolerated in the treatment of most noninfectious uveitis. On the rate of intraocular inflammation control, one multicenter study (Vallet, 2015) yielded similar results in the response rates to infliximab in uveitis, which were 87 and 93% at 6 and 12 months. Comparatively, our study contained data from more studies, larger sample size (426 *vs*. 77), wider range of age, and broader diversity of noninfectious uveitis and, therefore, might have a better population representation.

### Limitations

This study has some limitations. First, the included studies had various uveitis etiologies such as JIA, Behcet’s disease, sarcoidosis, and spondyloarthropathy-associated uveitis. Owing to the limited number of studies and paucity of clinical data, we did not conduct subgroup analysis according to basic disease, although clinical physicians might be interested in IFX efficacy for uveitis of different etiologies. Second, it could cause bias when the study included refractory noninfectious uveitis related to seronegative spondyloarthropathy or juvenile idiopathic arthritis. Limited by the included studies, we did not have access to individual patient data with refractory noninfectious uveitis related to seronegative spondyloarthropathy or JIA, so we could not eliminate these patients. Third, the proportion of corticosteroid-sparing, IFX discontinuation, and observed AEs were simply analyzed by averages without considering the weight of each study. Finally, in view of the fact that most studies used for analysis only had evidence strengths of Oxford Centre for Evidence-Based Medicine 2b, 3a, and 4, a dialectic point of view is still needed when referring to these results.

## Conclusion

This meta-analysis revealed the overall efficacy of IFX in the treatment of refractory noninfectious uveitis and demonstrated its good effects in short-term and long-term intraocular inflammation control, VA improvement, and corticosteroid-sparing. Meanwhile, we presented the common adverse events in the uveitis treatment with IFX, which may provide a reference for future prevention of adverse events. More high-quality, large-scale clinical trials with a similar design are needed to further prove the efficacy of IFX in the treatment of uveitis with stronger evidence.

## Data Availability

The original contributions presented in the study are included in the article/Supplementary Material; further inquiries can be directed to the corresponding author.
