# Supplementary figures and images for: The Efficacy and Safety of Infliximab in Refractory Noninfectious Uveitis: A Meta-Analysis of Observational Studies
Source: Front Pharmacol. 2021 Sep 16;12:620340. doi: 10.3389/fphar.2021.620340 (PMC8481770; doi:10.3389/fphar.2021.620340)

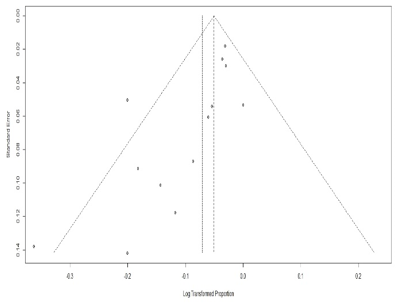

Supplement: Supplementary file 1 [file Image1.TIFF]
