# Supplementary material for: The Efficacy and Safety of Infliximab in Refractory Noninfectious Uveitis: A Meta-Analysis of Observational Studies
Source: Front Pharmacol. 2021 Sep 16;12:620340. doi: 10.3389/fphar.2021.620340 (PMC8481770; doi:10.3389/fphar.2021.620340)
Supplement: Supplementary file 2 [file Table1.DOCX]

**Table: Summary characteristics of the studies selected**

| **Study/ country** | **No. of patients (M/F)** | **Age, years** | **Aetiology** | **Follow-up** **duration (months)** | **CS at baseline** | **Inflammation control (n/N)** | **Visual acuity change** | **Medication tapering** | | **AEs** | **Treatment interruption** | **Evidence strength** |
| --- | --- | --- | --- | --- | --- | --- | --- | --- | --- | --- | --- | --- |
| Sharma, Ramanan ^42^ /UK | 6(NA) | Median14(8-18) | juvenile onset rheumatological disease =6 | ≥ 6(6-15) | NA | 5/6 at 6 months | Controlled: 4/12 eyes (4/6 patients).  No absence of any worsening: 11/12 eyes (5/6 patients) | | 3/6 patients reduce to 5 mg/day | No SAEs.  MAE: 1/6 patients developed new psoriasis. | 2/6 patients stopped IFX, 1 for remission, 1 due to treatment failure | Level 4 |
| Martel, Esterberg ^37^ /USA | 31 (11/20) | Median25.3 | ankylosing spondylitis =2, reactive arthritis= 2, psoriatic arthritis =3, JIA =5, Behçet = 6, Vogt-Koyanagi-Harada syndrome =2, sarcoidosis =3 | 12 | 61.3% | 23/28 at 6 months,  16/23 at 12 months | NA | | 14/23 of cases reducing prednisone to < 10 mg daily dose | SAEs (2/31): pulmonary infection (n=1) and bronchial spasm (n=1).  MAEs (1/31): myalgias, rash, nausea, lupus-like syndrome (n=1). | 10/31 patients stopped IFX. 2 for SAEs, 3 for remission,  5 for treatment-unrelated reasons | Level 4 |
| Yalcindag and Kose ^49^ /Turkey | 20  (13/7) | Mean  27.9 ± 4.2 | Behçet =20 | ≥12 | NA | 16/20 | VA (logMAR): Pre-treatment:  1.26 ± 0.18,  Month 12:  0.65 ± 0.14  · | | NA | SAEs (3/20):  direct infusion reaction (n=1), drug-induced lupus (n=1),  genital warts (n=1).  MAEs (1/20): psoriasiform rash (n=1). | 10/20 patients  stopped IFX. 3 for SAEs, 5 for remission, 2 for  lost to follow-up | Level 4 |
| Mercier, Ribeiro ^38^ /France | 13(NA) | NA | Behçet =7, birdshot =3, sarcoidosis =2, idiopathic =8, sympathetic ophthalmia =1  (Some patients used other TNF-α) | 31.7±13.1  (13-67) | Local CS: 80.9%  Oral CS: 76.2% | 12/12 at 6 months,  11/12 at 12 months | Unextractable | | NA | SAEs (1/13):  severe infectious (n=1).  MAEs (3/13):  pseudofolliculitis (n=3). | 1/13 patients discontinued the due to SAEs. | Level 4 |
| Sobrin, Kim ^45^ /USA | 27  (18/9) | Mean  37.3(6-66) | rheumatoid arthritis =2, ankylosing spondylitis =2, Behcet =6, JIA =5, reactive arthritis =2, relapsing polychondritis =1, Crohn disease =1, psoriasis =1, mucous membrane pemphigoid =1 | Mean:  25.6 | 30% | 20/24 | Controlled: 9/48 eyes (7/24 patients)  No absence of any worsening: 46/48 eyes (22/24 patients). (at ≥12months) | | NA | SAEs: 1/27 patient developed a lupus-like reaction. MAEs: 2/27 patients noted headache and flushing. | 1/27 patients discontinued IFX due to SAEs. | Level 4 |
| Tugal-Tutkun, Ayranci ^46^ / Turkey | 20  (13/7) | Mean  7.1(3-12) | idiopathic pars planitis =8, JIA =7, idiopathic chronic =4, Behçet =1 | Median:  30 | Systemic CS: 75%  Topical CS: 100% | 19/20  (<6 month);  9/11 at 12 months | Controlled: 7/22 eyes (7/11 patients) No absence of any worsening: 22/22 eyes (11/11 patients). (at ≥12months) | | 20/20 of patients discontinued topical CS; 14/15 of patients discontinued systemic CS. | No SAEs.  5/20 patients were found to have MAEs due to mild infusion reaction (n=1), herpes labialis (n=1), sinusitis (n=1), cutaneous herpes zoster (n=2) | NO discontinuation  due to SAEs. | Level 4 |
| Pichaporn, Ofelya ^40^ /UK | 22  (5/17) | Median  53(29-74) | birdshot =22 | 12 | 100% | 9/11 at 6 months, 8/9 at 12 months | 84.1%, 91.7% and 96.7% of eyes had a VA of 20/40 or better at 0, 6 and 12 months. | | 18/18 and 15/15 at 6 and 12 months were able to taper prednisone to ≤10 mg/day. | SAEs (6/22): allergic reactions (n=2), muscle weakness, myalgia, and paresthesia (n=1), lupus-like reactions (n=2), opportunistic fungal skin infection (n=1) | 9/22 of patients discontinued IFX for ineffectiveness (n=1), other reasons (n=2) and SAEs (n=6). | Level 4 |
| Fan ^50^ /China | 9 | NA | spondyloarthritis =9 | ≥ 6 | NA | 8/9  (< 6 month) | Controlled: 6/9 patients | | NA | NA | NA | Level 4 |
| Simonini, Taddio ^43^ /USA | 17  (7/10) | Median  10.4 (5.2- 13.10) | JIA =10, idiopathic =5, sarcoidosis =1, Behçet =1 | ≥12 | Oral CS:  100% | 16/17 at 12 months | Controlled: 20/32 eyes (13/16 patients) | | 16/17 of patients were able to stop CS treatment. | SAE (1/17): severe infusion reaction (n=1). MAEs (2/17): leukopenia (n=1), liver enzymes increased (n=1) | 1/17 patients discontinued IFX due to SAEs. | Level 2b |
| Kruh, Yang ^36^ /USA | 88 (20/68) | NA | idiopathic =34,  JIA =16,  birdshot =13,  HLA-B27 spondyloarthropathy =6,  sarcoidosis =6,  Behçet =3 | Mean:  28.75 | Local CS:20.5% Systemic CS:11.4 % | 72/88 | NA | | 22/22  (6/6 patients stopped intravenous methylprednisolone; 10/16 patients stopped oral CS; The mean dose of oral CS for 6/16 patients continuing at 6 months was 4.6 mg/day) | SAEs (17/88): rash (n=4), increased liver function test (n=2), autoimmune hepatitis (n=1), infusion-related reaction (n=3), fatigue (n=2), chronic infection (n=2), myalgia (n=1), drug-induced lupus (n=1). The most common MAEs (15/88) were skin rash (n=8) and fatigue (n=7) | 17/88 patients discontinued IFX due to SAEs. | Level 3a |
| Noy, Ujwala S ^39^ /USA | 13(5/8) | Mean  13(8-18) | neurosarcoidosis =1, JIA =5, HLA B27 spondyloarthropathy =1, none =7 | Mean 21 | 100% | 13/13 | NA | | 9/13 patients had achieved steroid-free remission. | No SAEs. | No discontinuation due to SAEs. | Level 2b |
| Giardina, Ferrante ^35^ /Italy | 19  (17/2) | Mean  33.3(18-53) | Behçet =19 | 12 | 100% | 18/19 | 18/19 patients improved 3 lines; 1/19 patients improved 2 line. | | NA | 1/19 patients were found to have SAEs due to non-Hodgkin lymphoma. The most common MAEs were headache, dizziness and tachycardia. | 3/19 of patients stopped treatment with IFX. One for personal reason (responder), one for SAEs and the last for lack of efficacy. | Level 4 |
| Sharma, Damato ^41^ /UK | 34(NA) | NA | idiopathic =19,  systemic vasculitis =1, ankylosing spondylitis =1,  Behçet =15 JIA =2,  juvenile sarcoidosis =1, psoriatic arthropathy =2,  sarcoidosis =1,  undifferentiated arthritis =1 (Some patients used other TNF-α) | Median  3.2 years | Oral CS:  93%  Local CS：  58.6% | 33/34 | Unextractable | | NA | SAEs: urinary tract infection (n=1) and significant cataract progression (n=1). MAEs: 2 patients exhibited transient elevation of the serum creatinine level. | No discontinuation due to SAEs. | Level 2b |
| Vallet, Riviere ^47^ /France | 77 (42/35) | Median  32(27-37) | Behçet =77 | Median  21 | 89.5% | 54/56 | NA | | NA | No SAEs. 20/77 patients were found to have MAEs due to Infections (n=11), Injection site reaction (n=4), Auto-immune disease (n=1), Demyelinating disease(n=1), Neoplasia (n=1). | No discontinuation due to SAEs. | Level 4 |
| Vallet, Seve ^48^ /France | 98(NA) | Median  31(21–42) | Behçet (36%),  JIA (22%),  sarcoidosis (6%), spondyloarthropathy (10%) | Median  36 | 84% | 95/98 | NA | | NA | SAE (15/98): infections (n=5), hypersensitivity reactions (n=5), autoimmune diseases (n=3), neoplasia (n=2). | NA | Level 4 |
| Simonini, Zannin ^44^ /  USA | 15  (5/10) | Median  12(5-21) | idiopathic =3, JIA =10, Behçet =1, sarcoidosis=1 | Median  30(16-38) | Oral CS:  100% | 13/15  (≤6 months),  13/15 at 12 months | Controlled: 16/26 eyes (10/13 patients)  No absence of any worsening 25/26 eyes (12/13 patients) | | 13/15 patients were able to stop CS treatment within 3 months. | SAE (1/15): severe infusion reaction (n=1),  MAEs (2/15): episode of leucopenia (n=1), liver enzymes increased by 3-fold (n=1) | 1/15 patients discontinued IFX due to SAEs. | Level 4 |
| **Notes:** Continuous variables are shown as the mean ± SD or median (range).  **Abbreviations**: IFX, infliximab; CS, corticosteroid; JIA, juvenile idiopathic arthritis; AEs, adverse events; SAEs, serious adverse events; MAEs, minor adverse events; BCVA, best-corrected visual acuity; VA, visual acuity; NA, not available. | | | | | | | | | | | | |
