# Supplementary material for: The Efficacy and Safety of Infliximab in Refractory Noninfectious Uveitis: A Meta-Analysis of Observational Studies
Source: Front Pharmacol. 2021 Sep 16;12:620340. doi: 10.3389/fphar.2021.620340 (PMC8481770; doi:10.3389/fphar.2021.620340)

Table S1: Summary characteristics of the studies selected

Study/ country	No. of patients (M/F)	Age, years	Aetiology	Follow-up duration (months)	CS at baseline	Inflammation control (n/N)	Visual acuity change	Medication tapering	AEs	Treatment interruption	Evidence strength
Sharma, Ramanan [42] /UK	6(NA)	Median 14(8-18)	juvenile onset rheumatological disease =6	≥ 6(6-15)	NA	5/6 at 6 months	Controlled: 4/12 eyes (4/6 patients). No absence of any worsening: 11/12 eyes (5/6 patients)	3/6 patients reduce to 5 mg/day	No SAEs. MAE: 1/6 patients developed new psoriasis.	2/6 patients stopped IFX, 1 for remission, 1 due to treatment failure	Level 4
Martel, Esterberg [37] /USA	31 (11/20)	Median 25.3	ankylosing spondylitis =2, reactive arthritis= 2, psoriatic arthritis =3, JIA =5, Behçet = 6, Vogt-Koyanagi-Harada syndrome =2, sarcoidosis =3	12	61.3%	23/28 at 6 months, 16/23 at 12 months	NA	14/23 of cases reducing prednisone to < 10 mg daily dose	SAEs (2/31): pulmonary infection (n=1) and bronchial spasm (n=1). MAEs (1/31): myalgias, rash, nausea, lupus-like syndrome (n=1).	10/31 patients stopped IFX. 2 for SAEs, 3 for remission, 5 for treatment-unrelated reasons	Level 4
Yalcindag and Kose [49] /Turkey	20 (13/7)	Mean 27.9 ± 4.2	Behçet =20	≥12	NA	16/20	VA (logMAR): Pre-treatment: 1.26 ± 0.18, Month 12: 0.65 ± 0.14	NA	SAEs (3/20): direct infusion reaction (n=1), drug-induced lupus (n=1),	10/20 patients stopped IFX. 3 for SAEs, 5 for remission, 2 for	Level 4

							·		genital warts (n=1).<br>MAEs (1/20): psoriasiform rash (n=1).	lost to follow-up	
Mercier, Ribeiro [38] /France	13(NA)	NA	Behçet =7, birdshot =3, sarcoidosis =2, idiopathic =8, sympathetic ophthalmia =1 (Some patients used other TNF-α)	31.7±13.1 (13-67)	Local CS: 80.9%<br>Oral CS: 76.2%	12/12 at 6 months, 11/12 at 12 months	Unextractable	NA	SAEs (1/13): severe infectious (n=1).<br>MAEs (3/13): pseudofolliculitis (n=3).	1/13 patients discontinued the due to SAEs.	Level 4
Sobrin, Kim [45] /USA	27 (18/9)	Mean 37.3(6-66)	rheumatoid arthritis =2, ankylosing spondylitis =2, Behcet =6, JIA =5, reactive arthritis =2, relapsing polychondritis =1, Crohn disease =1, psoriasis =1, mucous membrane pemphigoid =1	Mean: 25.6	30%	20/24	Controlled: 9/48 eyes (7/24 patients)<br>No absence of any worsening: 46/48 eyes (22/24 patients). (at ≥12months)	NA	SAEs: 1/27 patient developed a lupus-like reaction.<br>MAEs: 2/27 patients noted headache and flushing.	1/27 patients discontinued IFX due to SAEs.	Level 4
Tugal-Tutkun, Ayranci	20 (13/7)	Mean 7.1(3-12)	idiopathic pars planitis =8, JIA =7,	Median: 30	Systemic CS: 75%	19/20 (<6 month);	Controlled: 7/22 eyes (7/11 patients) No	20/20 of patients discontinue	No SAEs.<br>5/20 patients were found to have	NO discontinuation due to SAEs.	Level 4

[46] / Turkey			idiopathic chronic =4, Behçet =1		Topical CS: 100%	9/11 at 12 months	absence of any worsening: 22/22 eyes (11/11 patients). (at ≥12months)	d topical CS; 14/15 of patients discontinued systemic CS.	MAEs due to mild infusion reaction (n=1), herpes labialis (n=1), sinusitis (n=1), cutaneous herpes zoster (n=2)		
Pichaporn, Ofelya [40] /UK	22 (5/17)	Median 53(29-74)	birdshot =22	12	100%	9/11 at 6 months, 8/9 at 12 months	84.1%, 91.7% and 96.7% of eyes had a VA of 20/40 or better at 0, 6 and 12 months.	18/18 and 15/15 at 6 and 12 months were able to taper prednisone to ≤10 mg/day.	SAEs (6/22): allergic reactions (n=2), muscle weakness, myalgia, and paresthesia (n=1), lupus-like reactions (n=2), opportunistic fungal skin infection (n=1)	9/22 of patients discontinued IFX for ineffectiveness (n=1), other reasons (n=2) and SAEs (n=6).	Level 4
Fan [50] /China	9	NA	spondyloarthritis =9	≥ 6	NA	8/9 (< 6 month)	Controlled: 6/9 patients	NA	NA	NA	Level 4
Simonini, Taddio [43] /USA	17 (7/10)	Median 10.4 (5.2-13.10)	JIA =10, idiopathic =5, sarcoidosis =1, Behçet =1	⩾12	Oral CS: 100%	16/17 at 12 months	Controlled: 20/32 eyes (13/16 patients)	16/17 of patients were able to stop CS treatment.	SAE (1/17): severe infusion reaction (n=1). MAEs (2/17): leukopenia (n=1), liver	1/17 patients discontinued IFX due to SAEs.	Level 2b

									enzymes increased (n=1)		
Kruh, Yang [36] /USA	88 (20/68)	NA	idiopathic =34, JIA =16, birdshot =13, HLA-B27 spondyloarthropathy =6, sarcoidosis =6, Behçet =3	Mean: 28.75	Local CS:20.5 % Systemic CS:11.4 %	72/88	NA	22/22 (6/6 patients stopped intravenous methylprednisolone; 10/16 patients stopped oral CS; The mean dose of oral CS for 6/16 patients continuing at 6 months was 4.6 mg/day)	SAEs (17/88): rash (n=4), increased liver function test (n=2), autoimmune hepatitis (n=1), infusion-related reaction (n=3), fatigue (n=2), chronic infection (n=2), myalgia (n=1), drug-induced lupus (n=1). The most common MAEs (15/88) were skin rash (n=8) and fatigue (n=7)	17/88 patients discontinued IFX due to SAEs.	Level 3a
Noy, Ujwala S [39] /USA	13(5/8)	Mean 13(8-18)	neurosarcoidosis =1, JIA =5, HLA B27	Mean 21	100%	13/13	NA	9/13 patients had achieved	No SAEs.	No discontinuation due to SAEs.	Level 2b

			spondyloarthropathy =1, none =7					steroid-free remission.			
Giardina, Ferrante [35] /Italy	19 (17/2)	Mean 33.3(18 -53)	Behçet =19	12	100%	18/19	18/19 patients improved 3 lines; 1/19 patients improved 2 line.	NA	1/19 patients were found to have SAEs due to non-Hodgkin lymphoma. The most common MAEs were headache, dizziness and tachycardia.	3/19 of patients stopped treatment with IFX. One for personal reason (responder), one for SAEs and the last for lack of efficacy.	Level 4
Sharma, Damato [41] /UK	34(NA)	NA	idiopathic =19, systemic vasculitis =1, ankylosing spondylitis =1, Behçet =15 JIA =2, juvenile sarcoidosis =1, psoriatic arthropathy =2, sarcoidosis =1, undifferentiated arthritis =1 (Some patients used other TNF-α)	Median 3.2 years	Oral CS: 93% Local CS: 58.6%	33/34	Unextractable	NA	SAEs: urinary tract infection (n=1) and significant cataract progression (n=1). MAEs: 2 patients exhibited transient elevation of the serum creatinine level.	No discontinuation due to SAEs.	Level 2b

Vallet, Riviere [47] /France	77 (42/35)	Median 32(27-37)	Behçet =77	Median 21	89.5%	54/56	NA	NA	No SAEs. 20/77 patients were found to have MAEs due to Infections (n=11), Injection site reaction (n=4), Auto-immune disease (n=1), Demyelinating disease(n=1), Neoplasia (n=1).	No discontinuation due to SAEs.	Level 4
Vallet, Seve [48] /France	98(NA)	Median 31(21–42)	Behçet (36%), JIA (22%), sarcoidosis (6%), spondyloarthropathy (10%)	Median 36	84%	95/98	NA	NA	SAE (15/98): infections (n=5), hypersensitivity reactions (n=5), autoimmune diseases (n=3), neoplasia (n=2).	NA	Level 4
Simonini, Zannin [44] / USA	15 (5/10)	Median 12(5-21)	idiopathic =3, JIA =10, Behçet =1, sarcoidosis=1	Median 30(16-38)	Oral CS: 100%	13/15 (≤6 months), 13/15 at 12 months	Controlled: 16/26 eyes (10/13 patients) No absence of any worsening 25/26 eyes	13/15 patients were able to stop CS treatment within 3 months.	SAE (1/15): severe infusion reaction (n=1), MAEs (2/15): episode of leucopenia (n=1), liver enzymes	1/15 patients discontinued IFX due to SAEs.	Level 4

			(12/13 patients)	increased by 3-fold (n=1)

Notes: Continuous variables are shown as the mean ± SD or median (range).

Abbreviations: IFX, infliximab; CS, corticosteroid; JIA, juvenile idiopathic arthritis; AEs, adverse events; SAEs, serious adverse events; MAEs, minor adverse events; BCVA, best-corrected visual acuity; VA, visual acuity; NA, not available.

Quality assessment by agency for Healthcare Research and Quality (AHRQ)

Item	Yes	No	Unclear
1) Define the source of information (survey, record review)			
2) List inclusion and exclusion criteria for exposed and unexposed subjects (cases and controls) or refer to previous publications			
3) Indicate time period used for identifying patients			
4) Indicate whether or not subjects were consecutive if not population-based			
5) Indicate if evaluators of subjective components of study were masked to other aspects of the status of the participants			
6) Describe any assessments undertaken for quality assurance purposes (e.g., test/retest of primary outcome measurements)			
7) Explain any patient exclusions from analysis			
8) Describe how confounding was assessed and/or controlled			
9) If applicable, explain how missing data were handled in the analysis			
10) Summarize patient response rates and completeness of data collection			
11) Clarify what follow-up, if any, was expected and the percentage of patients for which incomplete data or follow-up was obtained			

Table S2: Quality Assessment of Inclusion Studies

study	item 1	item 2	item 3	item 4	item 5	item 6	item 7	item 8	item 9	item 10	item 11	Sum of score
Simonini, Taddio [42]	1	0	1	1	1	1	1	1	0	0	1	8
Mercier, Ribeiro [37]	1	0	1	1	0	1	1	0	0	0	1	6
Yalcindag and Kose [49]	1	0	1	1	0	1	1	1	0	0	1	7
Noy, Ujwala S [38]	1	0	1	1	0	1	1	0	0	0	1	6
Tugal-Tutkun, Ayranci [45]	1	0	1	1	0	1	1	0	0	0	0	5
Vallet, Seve [46]	1	0	1	1	0	1	1	1	0	0	1	7
Sharma, Damato [40]	1	0	1	1	0	1	1	1	0	0	1	7
Fan [50]	1	0	1	1	0	1	1	1	0	0	1	7
Simonini, Zannin [43]	1	0	1	1	0	1	1	1	0	0	1	7
Martel, Esterberg [36]	1	0	1	1	0	1	1	0	0	0	1	6
Pichaporn, Ofelya [39]	1	0	1	1	0	1	1	0	0	0	1	6
Kruh, Yang [35]	1	0	1	1	0	1	1	0	0	0	1	6
Sharma, Ramanan [41]	1	0	0	1	0	0	1	0	0	0	0	3
Fan [47]	1	0	1	1	0	1	1	0	0	0	0	5
Vallet, Seve [48]	1	0	1	1	0	1	1	1	0	0	1	7
Sobrin, Kim [44]	1	0	1	1	0	1	1	0	0	0	1	6

Figure S1: Funnel plots for publication bias

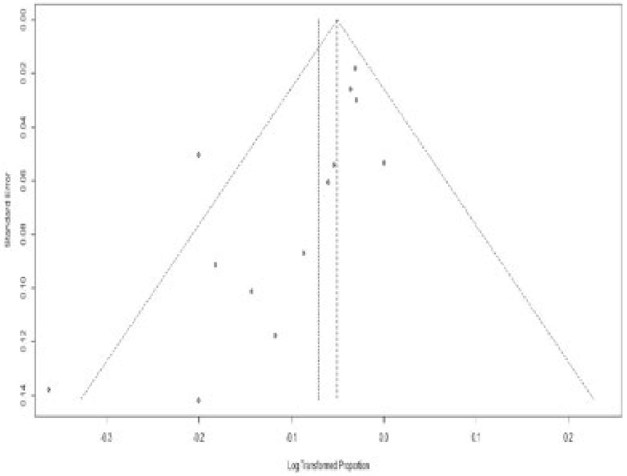

Supplement: Supplementary file 3 [file DataSheet1.zip › Supplementary Material.pdf]
