# Supplementary material for: The Efficacy and Safety of Infliximab in Refractory Noninfectious Uveitis: A Meta-Analysis of Observational Studies
Source: Front Pharmacol. 2021 Sep 16;12:620340. doi: 10.3389/fphar.2021.620340 (PMC8481770; doi:10.3389/fphar.2021.620340)
Supplement: Supplementary file 4 [file Table2.DOCX]

| **Item** | **Yes** | **No** | **Unclear** |
| --- | --- | --- | --- |
| 1) Define the source of information (survey, record review) |  |  |  |
| 1. List inclusion and exclusion criteria for exposed and unexposed subjects (cases and controls) or refer to previous publications |  |  |  |
| 1. Indicate time period used for identifying patients |  |  |  |
| 1. Indicate whether or not subjects were consecutive if not population-based |  |  |  |
| 1. Indicate if evaluators of subjective components of study were masked to other aspects of the status of the participants |  |  |  |
| 1. Describe any assessments undertaken for quality assurance purposes (e.g., test/retest of primary outcome measurements) |  |  |  |
| 1. Explain any patient exclusions from analysis |  |  |  |
| 1. Describe how confounding was assessed and/or controlled |  |  |  |
| 9) If applicable, explain how missing data were handled in the analysis |  |  |  |
| 10) Summarize patient response rates and completeness of data collection |  |  |  |
| 11）Clarify what follow-up, if any, was expected and the percentage of patients for which incomplete data or follow-up was obtained |  |  |  |

**Quality assessment by agency for Healthcare Research and Quality (AHRQ)**

| **study** | **item**  **1** | **item 2** | **item 3** | **item 4** | **item 5** | **item 6** | **item 7** | **item 8** | **item 9** | **item 10** | **item 11** | **Sum of score** |
| --- | --- | --- | --- | --- | --- | --- | --- | --- | --- | --- | --- | --- |
| **Simonini, Taddio[42]** | **1** | **0** | **1** | **1** | **1** | **1** | **1** | **1** | **0** | **0** | **1** | **8** |
| **Mercier, Ribeiro[37]** | **1** | **0** | **1** | **1** | **0** | **1** | **1** | **0** | **0** | **0** | **1** | **6** |
| **Yalcindag and Kose [49]** | **1** | **0** | **1** | **1** | **0** | **1** | **1** | **1** | **0** | **0** | **1** | **7** |
| **Noy, Ujwala S[38]** | **1** | **0** | **1** | **1** | **0** | **1** | **1** | **0** | **0** | **0** | **1** | **6** |
| **Tugal-Tutkun, Ayranci[45]** | **1** | **0** | **1** | **1** | **0** | **1** | **1** | **0** | **0** | **0** | **0** | **5** |
| **Vallet, Seve[46]** | **1** | **0** | **1** | **1** | **0** | **1** | **1** | **1** | **0** | **0** | **1** | **7** |
| **Sharma, Damato[40]** | **1** | **0** | **1** | **1** | **0** | **1** | **1** | **1** | **0** | **0** | **1** | **7** |
| **Fan [50]** | **1** | **0** | **1** | **1** | **0** | **1** | **1** | **1** | **0** | **0** | **1** | **7** |
| **Simonini, Zannin[43]** | **1** | **0** | **1** | **1** | **0** | **1** | **1** | **1** | **0** | **0** | **1** | **7** |
| **Martel, Esterberg[36]** | **1** | **0** | **1** | **1** | **0** | **1** | **1** | **0** | **0** | **0** | **1** | **6** |
| **Pichaporn, Ofelya[39]** | **1** | **0** | **1** | **1** | **0** | **1** | **1** | **0** | **0** | **0** | **1** | **6** |
| **Kruh, Yang[35]** | **1** | **0** | **1** | **1** | **0** | **1** | **1** | **0** | **0** | **0** | **1** | **6** |
| **Sharma, Ramanan[41]** | **1** | **0** | **0** | **1** | **0** | **0** | **1** | **0** | **0** | **0** | **0** | **3** |
| **Fan[47]** | **1** | **0** | **1** | **1** | **0** | **1** | **1** | **0** | **0** | **0** | **0** | **5** |
| **Vallet, Seve [48]** | **1** | **0** | **1** | **1** | **0** | **1** | **1** | **1** | **0** | **0** | **1** | **7** |
| **Sobrin, Kim[44]** | **1** | **0** | **1** | **1** | **0** | **1** | **1** | **0** | **0** | **0** | **1** | **6** |

**Table 2: Quality Assessment of Inclusion Studies**
